# Surgical Management of Young Women with High-Risk Breast Cancer Receiving Neoadjuvant Systemic Therapy on the I-SPY2 Trial

**DOI:** 10.1245/s10434-026-20153-x

**Published:** 2026-08-03

**Authors:** J. Tseng, K. Dimitroff, C. A. M. Sauder, A. J. Chien, K. A. Ahmed, L. F. Allen, C. A. Arciero, E. E. Burke, J. M. Chandler, J. E. Jordan, M. Golshan, L. A. Gutnik, N. T. Jaskowiak, K. C. Hewitt, M. Howard-McNatt, F. Ito, I. M. Kang, S. B. Kesmodel, H. M. Kuerer, M. C. Lee, M. S. Moran, L. M. Postlewait, N. D. Prionas, R. Rao, C. R. Reyna, A. Schwer, N. K. Taunk, J. Tchou, A. M. Wallace, T. J. Li, J. Perlmutter, A. M. DeMichele, D. Yee, N. M. Hylton, L. J. van’t Veer, H. S. Rugo, W. F. Symmans, R. A. Shatsky, C. J. D. Isaacs, C. Yau, L. J. Esserman, J. C. Boughey, R. A. Mukhtar

**Affiliations:** 1 City of Hope Comprehensive Cancer Center, Irvine, CA USA; 2 Quantum Leap Healthcare Collaborative, San Francisco, CA USA; 3 University of California Davis, Sacramento, CA USA; 4 University of California San Francisco, San Francisco, CA USA; 5 H. Lee Moffitt Cancer Center and Research Institute, Tampa, FL USA; 6 Atrium Health Wake Forest Baptist, Winston-Salem, NC USA; 7 Emory University, Atlanta, GA USA; 8 The Ohio State University Health System, Columbus, OH USA; 9 Yale University School of Medicine, New Haven, CT USA; 10 University of Alabama at Birmingham, Birmingham, AL USA; 11 The University of Chicago Medicine, Chicago, IL USA; 12 University of Utah Health, Salt Lake City, UT USA; 13 University of Southern California, Los Angeles, CA USA; 14 University of Miami Hospital, Miami, FL USA; 15 UT MD Anderson Cancer Center, Houston, TX USA; 16 Columbia University Medical Center, New York, NY USA; 17 Loyola University Medical Center, Maywood, IL USA; 18 University of Pennsylvania, Philadelphia, PA USA; 19 University of California San Diego, San Diego, CA USA; 20 Gemini Group Consultancy, St. Louis, MO USA; 21 University of Minnesota, Minneapolis, MN USA; 22 City of Hope Comprehensive Cancer Center, Duarte, CA USA; 23 Georgetown University, Washington, DC USA; 24 Mayo Clinic, Rochester, MN USA

**Keywords:** Young women, Breast cancer, High risk, Surgery, Mastectomy, Breast conserving surgery

## Abstract

**Background:**

Mastectomy rates in women with breast cancer are higher in younger women than in older women. The impact of this more extensive surgery on overall survival (OS) and locoregional recurrence in younger women is unknown, especially after neoadjuvant systemic therapy (NST). This study evaluated surgical management and outcomes of patients aged ≤ 45 versus > 45 years enrolled in multicenter NST clinical trial, I-SPY2.0 (NCT01042379, PMID 37325931).

**Methods:**

We conducted a secondary data analysis comparing locoregional treatment in patients aged ≤ 45 versus > 45 years with clinical or molecular high-risk clinical stage II–III breast cancer treated from April 2010 to June 2022. Multivariate Cox proportional hazards models were used to evaluate associations between type of breast surgery with OS and locoregional recurrence-free interval by age group and tumor receptor subtype.

**Results:**

Of 1737 patients, 698 (40.2%) were aged ≤ 45 years. There were no significant differences in patient or tumor characteristics or residual cancer burden distribution between age groups. Although breast-conserving surgery was significantly less common in younger women (36.8% vs 48.5%, *p* < 0.001), surgery type was not associated with OS or locoregional recurrence-free interval for patients aged ≤ 45 years.

**Conclusions:**

Greater extent of breast surgery was not associated with improved outcomes in women aged ≤ 45 years. Choice of surgical procedure in the management of breast cancer is multifactorial, but young age alone does not warrant mastectomy following NST.

Despite breast-conservation therapy (BCT) having similar oncologic outcomes to mastectomy for many patients with early-stage breast cancer, mastectomy rates continue to be higher in younger women than in older women.^1–8^ The choice of mastectomy or BCT is guided by many factors, including tumor-to-breast ratio, patient concern for recurrence, family history, and high-risk germline pathogenic variants. Young age has also been historically considered as a potential reason to consider more extensive breast surgery. The causes for and impact of this more extensive breast surgery on overall survival and locoregional recurrence in younger women is relatively unknown, especially in the setting of surgery after neoadjuvant systemic therapy (NST).^9^ The goal of this study was to evaluate the trends and outcomes in the surgical management of patients aged ≤ 45 versus > 45 years enrolled in the I-SPY2.0 clinical trial (NCT01042379, PMID 37325931), a multicenter NST adaptive platform trial.

We have previously shown that BCT was not associated with worse locoregional outcomes than mastectomy, even in this high-risk I-SPY2.0 trial population receiving NST.^10^ Instead, response to therapy as reflected by residual cancer burden (RCB) was a significant predictor of recurrence, regardless of type of breast surgery performed.^10,11^ However, data on the optimal surgery approach for the breast and axilla in younger women are sparse, with most studies demonstrating high rates of mastectomy in younger than in older women. Here, we evaluated differences in breast surgery in women aged ≤ 45 years compared with those aged > 45 years, and the association between type of breast and axillary surgery and oncologic outcomes, stratified by tumor receptor subtype and response to therapy.

## Methods

Clinical and pathological factors were evaluated comparing patients aged ≤ 45 versus > 45 years at the time of diagnosis with clinical stage II–III breast cancer enrolled in the I-SPY2.0 clinical trial from April 2010 through June 2022. I-SPY2.0 is a prospective, randomized, adaptive platform study testing novel therapies in the neoadjuvant setting, with the rate of pathologic complete response at surgery being the primary endpoint. Patients are randomized to various treatment arms based on tumor characteristics, including receptor subtype; for I-SPY2.0, all patients undergo testing with a 70-gene assay (MammaPrint, Agendia, Irvine, CA, USA). MammaPrint is a 70-gene messenger RNA expression signature that is approved by the US Food and Drug Administration to estimate prognosis of early-stage breast cancer and aid in treatment selection.^12^ Patients undergo serial breast imaging with magnetic resonance imaging on clinical trial. Of note, the type of breast surgery performed is not mandated by the clinical trial but rather reflects surgical recommendations and patient choice.

The age cutoff of 45 years was chosen to align with other planned analyses looking at outcomes for young women on the I-SPY2.0 clinical trial. A subgroup analysis of women aged ≤ 40 years was also performed. Surgical therapy of the breast was categorized as breast-conserving surgery (BCS) or mastectomy (including total mastectomy, skin-sparing mastectomy, and nipple-sparing mastectomy). Axillary surgery was classified as sentinel lymph node (SLN) surgery or axillary lymph node dissection (ALND), with patients who had both SLN surgery and ALND categorized in the ALND group. Rates of nodal conversion from clinical node-positive to pathologic node-negative status and vice versa were also reviewed. Germline genetic mutation results and data on adjuvant radiation and adjuvant systemic therapy administration were not completely collected at the time of this study and were therefore unavailable for analysis.

To compare associations between continuous baseline characteristics and age groups, the Wilcoxon rank-sum test was used. To compare associations between categorical baseline characteristics and age groups, Pearson's Chi-squared test and Fisher's exact test were used. Tumor receptor subtype was categorized as hormone receptor positive (HR+), HER2 negative, triple negative (TNBC), or HER2 positive. HR+ breast cancer was defined as having estrogen receptor and/or progesterone receptor ≥5%. Associations between breast surgery and overall survival (OS) as well as local recurrence-free interval (LRFI) were examined stratified by age with the Kaplan–Meier method, log-rank test, and multivariate Cox proportional hazard regression models, adjusting for patient and clinical factors of tumor receptor subtype, clinical T category (T1/2 vs. T3/4), clinical nodal status (cN0 vs. cN+), histologic grade, axillary surgery type, and RCB classification.^11^ Statistical analyses were performed using R version 4.5.1. Two-tailed *p*-values < 0.05 were considered statistically significant.

## Results

### Patient and Tumor Characteristics

**Fig. 1:**
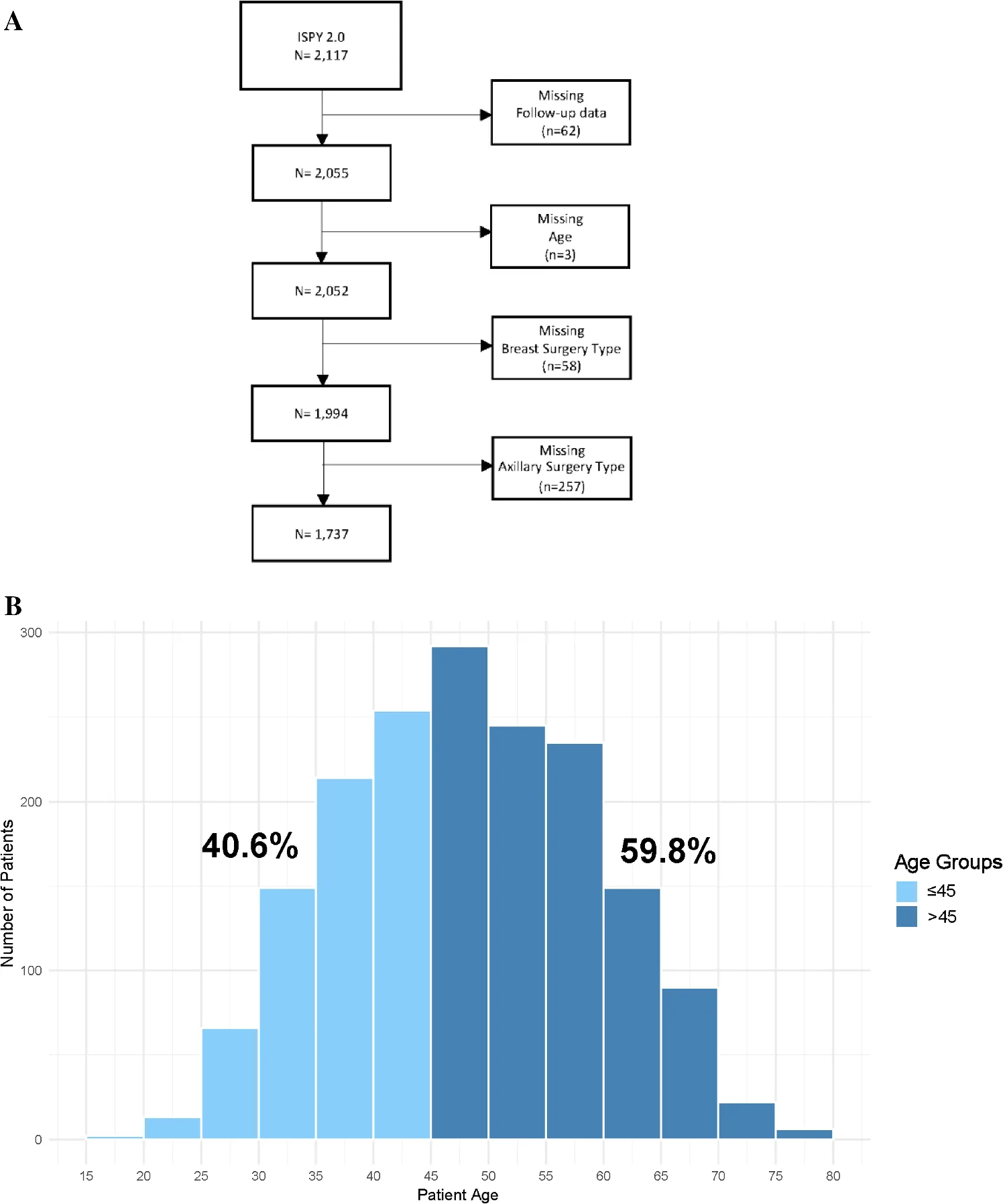
Study population and distribution. **A** Cohort diagram. **B** Population distribution by patient age.

In total, 1737 patients were included in this study, 698 (40.2%) aged ≤ 45 years and 1039 (59.8%) aged > 45 years (Fig. 1). There were no significant differences in patient characteristics of self-reported race/ethnicity or tumor features, including receptor subtype, clinical tumor/nodal categories, or histologic grade by age groups. RCB class distribution after NST was also similar between age groups (Table 1). Median follow-up time was 5.2 years, and 5-year LRFI was 91% in patients aged ≤ 45 years and 92% in patients aged > 45 years. The 5-year OS was 88% in both age groups.

**Table 1:** Patient and tumor characteristics by age groups

Characteristic	Age group		*p*-Value
	≤ 45 years (*n* = 698)	> 45 years (*n* = 1039)	
*Self-reported race*			0.084
White	536 (78.2%)	837 (81.6%)	
Black	80 (11.7%)	116 (11.3%)	
Asian/other	69 (10.1%)	73 (7.1%)	
*Ethnicity*			0.075
Not Hispanic	584 (84.3%)	900 (87.3%)	
Hispanic	109 (15.7%)	131 (12.7%)	
*Tumor subtype*			0.13
HR+HER2-	309 (44.3%)	441 (42.4%)	
TNBC	247 (35.4%)	344 (33.1%)	
HER2+	142 (20.3%)	254 (24.4%)	
*Histologic grade*			0.4
I/II	147 (29.1%)	230 (31.6%)	
III	358 (70.9%)	498 (68.4%)	
*Baseline clinical T category*			0.3
cT0-2	464 (67.7%)	716 (70.3%)	
cT3-4	221 (32.3%)	302 (29.7%)	
*Baseline clinical nodal status*			0.8
Negative	315 (46.3%)	471 (46.9%)	
Positive	365 (53.7%)	534 (53.1%)	
Number of total nodes removed (mean)	8.42 ± 8.55	8.40 ± 8.13	0.6
Number of positive lymph nodes removed	1.43 ± 3.63	1.35 ± 3.16	> 0.9
RCB index (mean)	1.53 (1.43)	1.52 (1.40)	> 0.9

Data are presented as mean ± standard deviation or n (%) unless otherwise indicated.

Wilcoxon rank sum test; Fisher’s exact test; Pearson’s Chi-squared test

HER2, human epidermal growth factor receptor 2; HR, hormone receptor; T, tumor; RCB, residual cancer burden; TNBC, triple-negative breast cancer.

### Breast Surgery

**Fig. 2:**
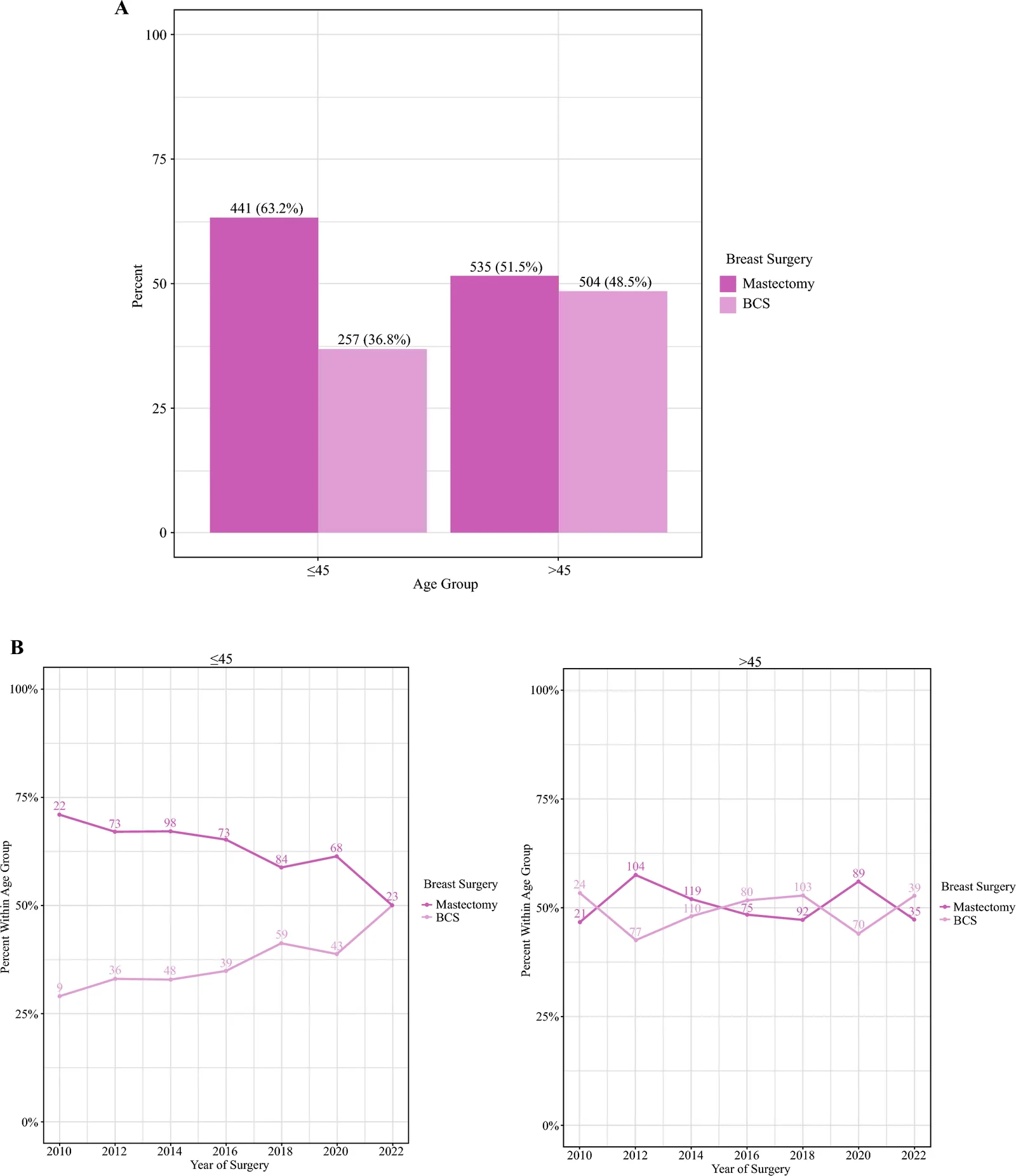

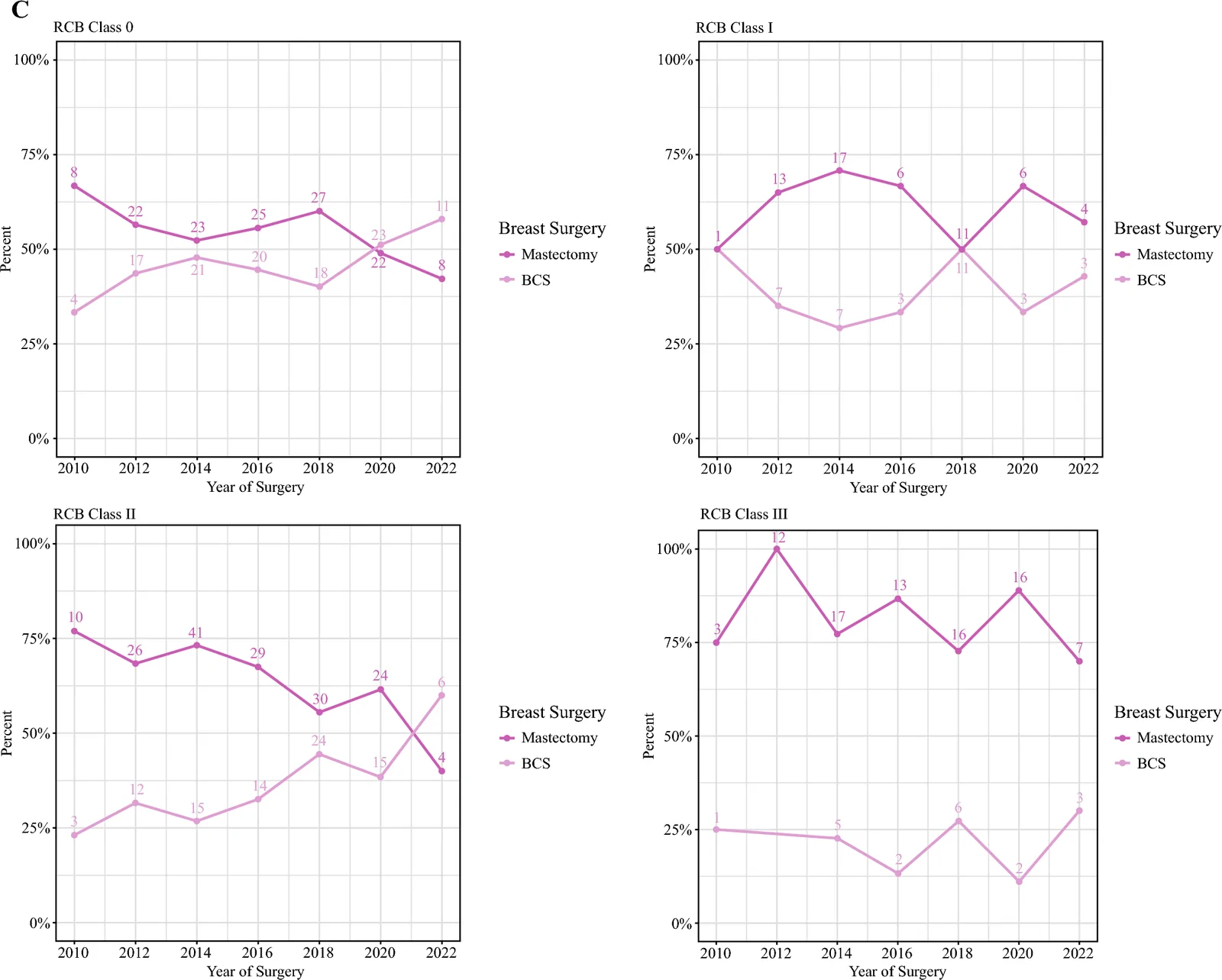
Breast surgery type **A** by age group, **B** over study time period (April 2010 to June 2022) by age groups and **C** by residual cancer burden (RCB) class. BCS, breast-conserving surgery.

Rates of BCS were significantly lower in those aged ≤ 45 years than in those > 45 years (36.8% vs 48.5%, *p* < 0.001) (Fig. 2A). However, there was an uptrend in the rates of BCS over the study period, increasing from 29% in 2010 to 50% in 2022 for patients aged ≤ 45 years (Fig. 2B). This increase in the rate of BCS over time occurred in patients with RCB class 0, I, and II disease, whereas patients aged ≤ 45 years with RCB class III disease had stable rates of BCS over time (Fig. 2C). When evaluating breast surgery type by age groups within tumor receptor subtypes, patients aged ≤ 45 years had higher rates of mastectomy than patients aged > 45 years within the HR+HER2- (66.0% vs 53.3%, *p* < 0.001) and the TNBC (60.3% vs 47.4%, *p* = 0.002) subtypes. There was no difference in breast surgery type by age within the HER2+ subtype (62.0% vs 53.9%, *p* = 0.12).

### Axillary Surgery

**Fig. 3:**
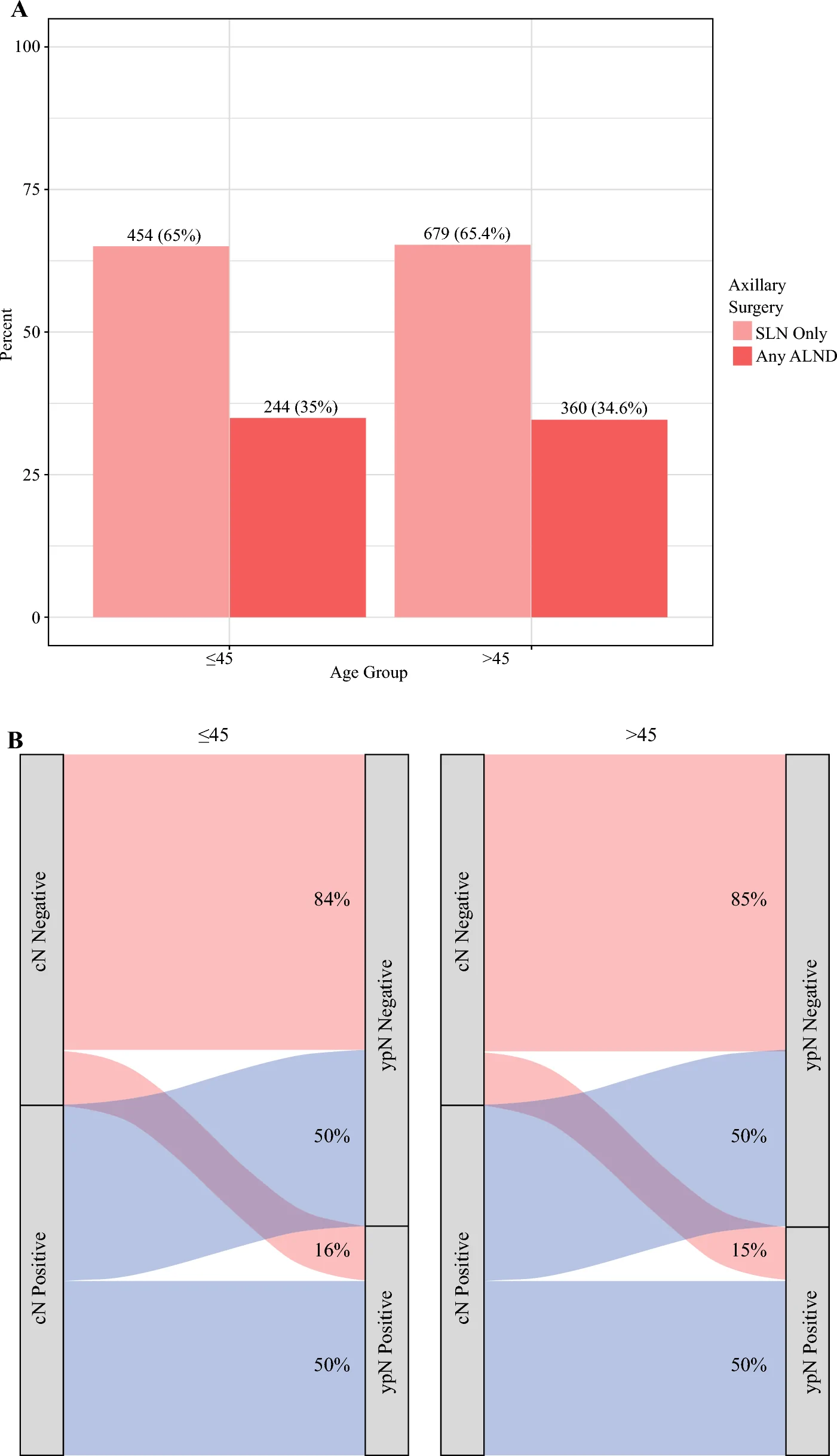
**A** Rates of axillary surgery and nodal conversion by age groups. **B** Sankey plot showing clinical to pathologic nodal conversion rates by age group. ALND, axillary lymph node dissection; SLN, sentinel lymph node.

There were no significant differences in extent of axillary surgery between those aged ≤ 45 years and those aged > 45 years (SLN surgery: 65.0% vs 65.4%; ALND: 35.0% vs 34.6%, *p* = 0.9) (Fig. 3A). When evaluating axillary surgery by age groups within tumor receptor subtypes, there was no significant difference in any subtype (HR + HER2-: *p* = 0.6, TNBC: *p* = 0.8, and HER2+: *p* = 0.5). The numbers of total lymph nodes removed at surgery and total positive lymph nodes removed at surgery were similar between the age groups (Table 1). The rates of nodal conversion (from clinical node positive to negative or negative to positive) were also similar for patients aged ≤ 45 years and those aged > 45 years (Fig. 3B).

### Overall Survival by Breast Surgery Type

**Fig. 4:**
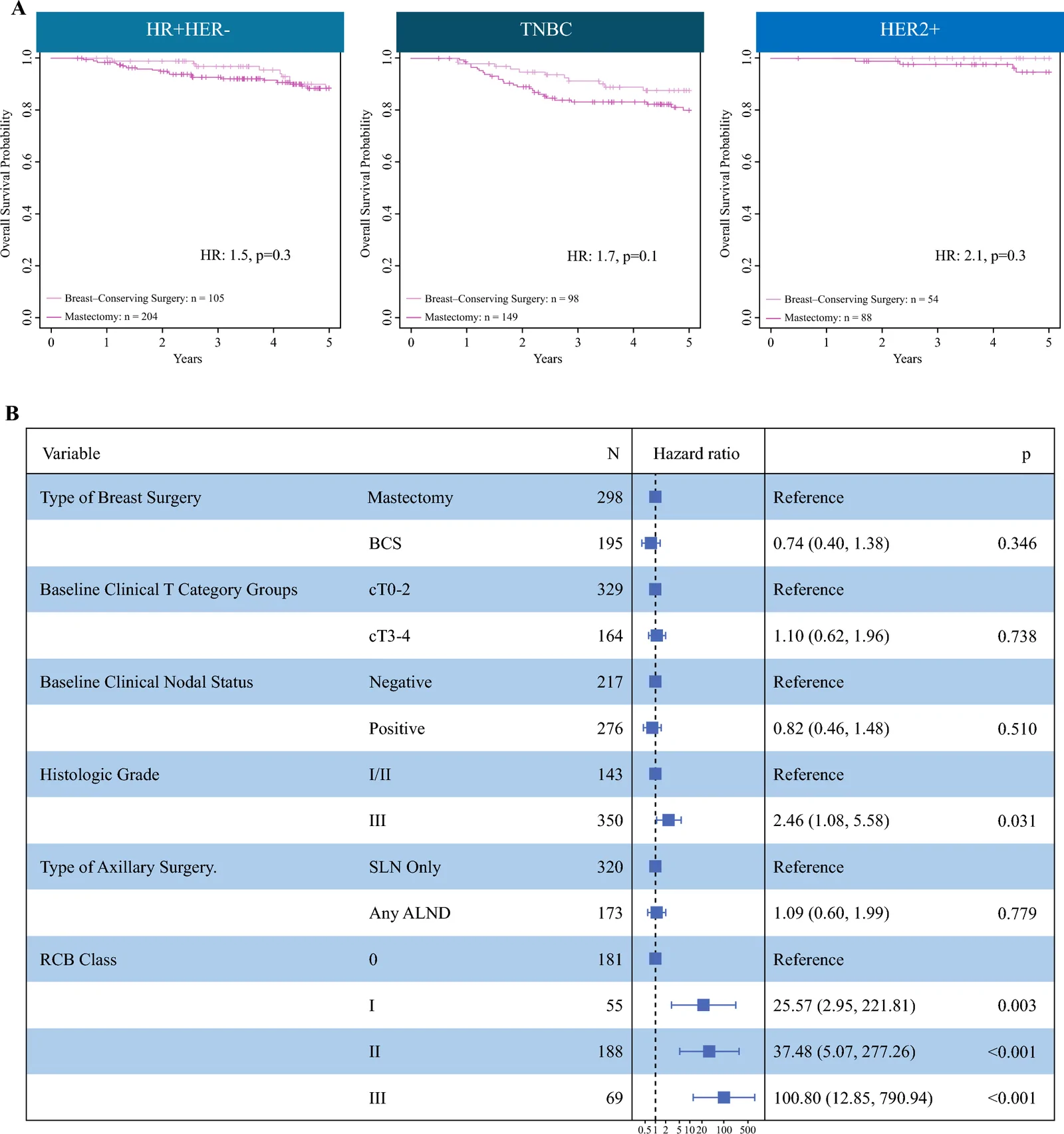
**A** Kaplan–Meier estimates of overall survival (OS) by breast surgery type across tumor subtypes and **B** forest plot depicting hazard ratios from Cox proportional hazards model for OS by breast surgery type and associated factors in patients aged ≤ 45 years. ALND, axillary lymph node dissection; BCS, breast-conserving surgery; HER2, human epidermal growth factor receptor 2; HR, hazard ratio; HR+, hormone receptor; RCB, residual cancer burden; SLN, sentinel lymph node surgery; T, tumor; TNBC, triple-negative breast cancer.

There was no significant difference in OS by breast surgery type across the tumor receptor subtypes (HR+HER2-: *p* = 0.3, TNBC: *p* = 0.1, and HER2+: *p* = 0.3) for patients aged ≤ 45 years (Fig. 4A). On multivariate analysis, there was no significant difference in OS by breast surgery type for patients aged ≤ 45 years (hazard ratio [HR] 0.74; 95% confidence interval [CI] 0.40–1.38; *p* = 0.346) (Fig. 4B). OS decreased with increasing histologic grade (HR 2.46; 95% CI 1.08–5.58; *p* = 0.031) and demonstrated a strong, stepwise association with higher RCB class (RCB I–III; HR range: 25.57–100.80; all *p* ≤ 0.003).

### Locoregional Recurrence-Free Interval by Breast Surgery Type

**Fig. 5:**
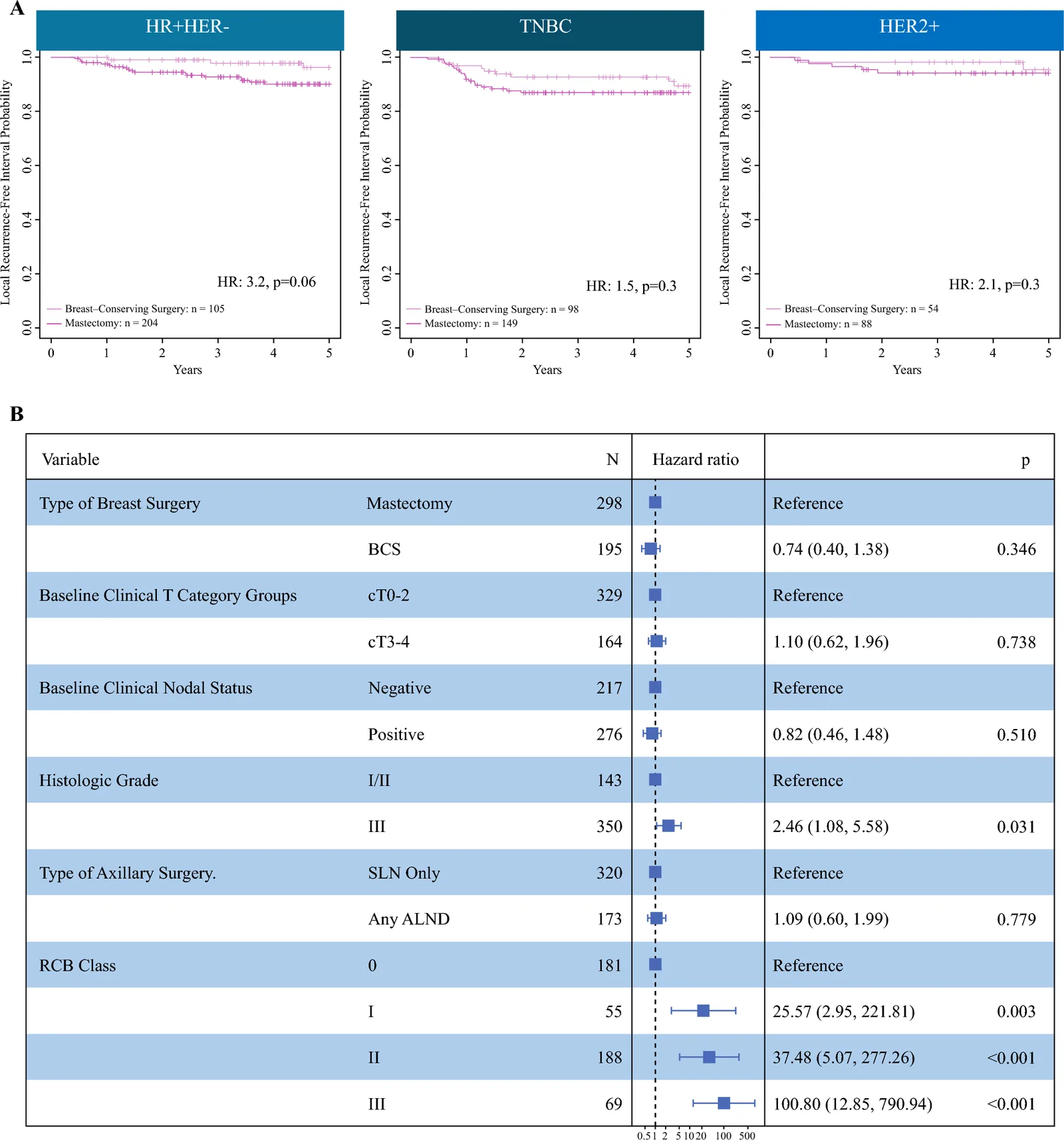
**A** Kaplan–Meier estimates of local recurrence-free interval (LRFI) by breast surgery type across tumor subtypes in patients aged ≤ 45 years. **B** Forest plot depicting hazard ratios (HRs) from Cox proportional hazards model for LRFI by breast surgery type and associated factors in patients aged ≤ 45 years. ALND, axillary lymph node dissection; BCS, breast-conserving surgery; HER2, human epidermal growth factor receptor 2; HR+, hormone receptor; RCB, residual cancer burden; SLN, sentinel lymph node surgery; T, tumor; TNBC, triple-negative breast cancer.

Similarly, for patients aged ≤ 45 years, there was no significant difference in LRFI by breast surgery type across the tumor receptor subtypes (HR+HER2-: *p* = 0.06, TNBC: *p* = 0.3, and HER2+: *p* = 0.3) (Fig. 5A). There was no significant difference in LRFI by breast surgery type (mastectomy vs. BCS) on multivariate analysis (HR 0.64; 95% CI 0.32–1.31; *p* = 0.222) (Fig. 5B). Factors influencing LRFI were higher histologic grade (HR 3.69; 95% CI 1.45–9.39; *p* = 0.006) and RCB class (I–III; HR 4.30–14.39; all p≤ 0.042).

### Patients Aged ≤ 40 Years

**Fig. 6:**
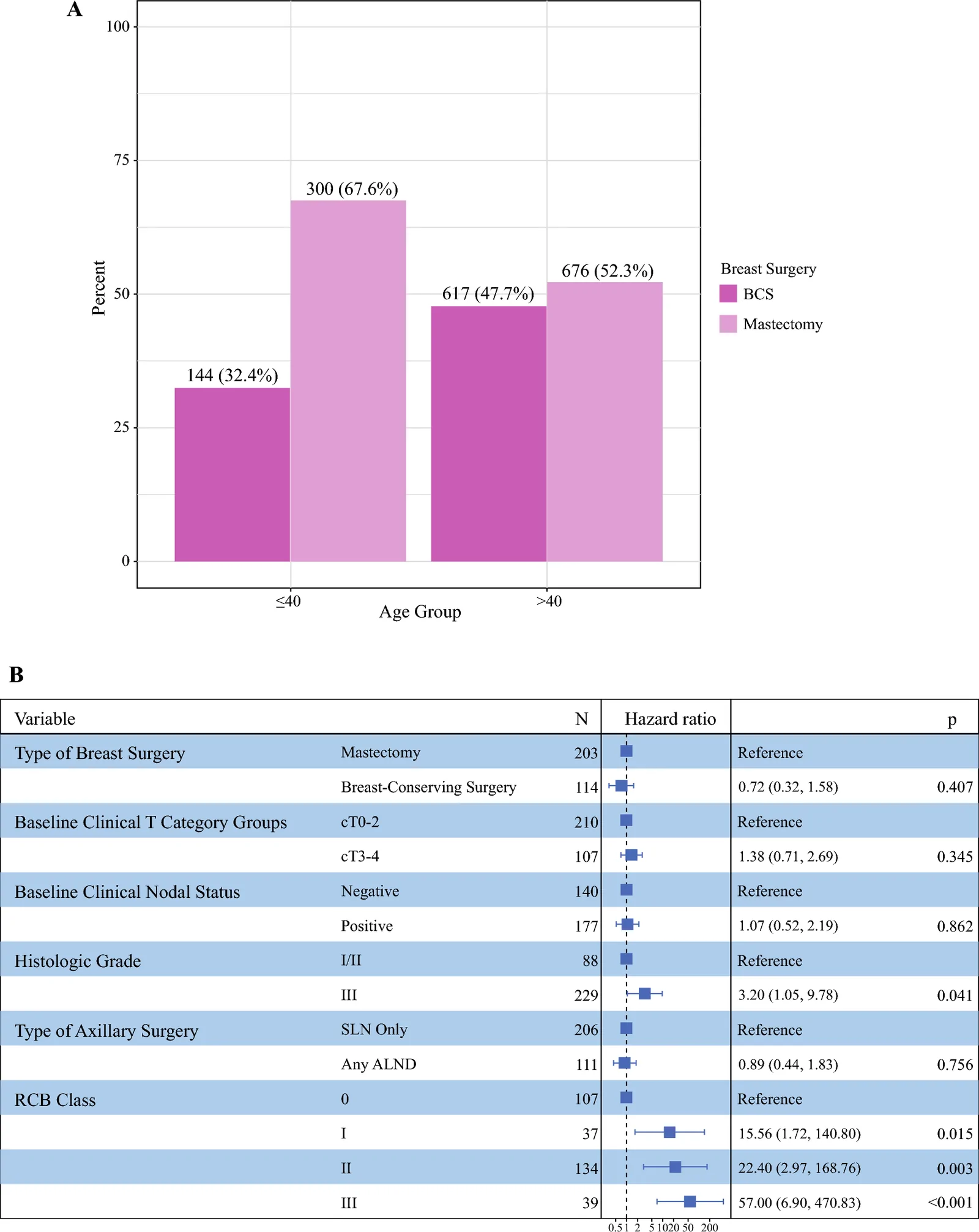

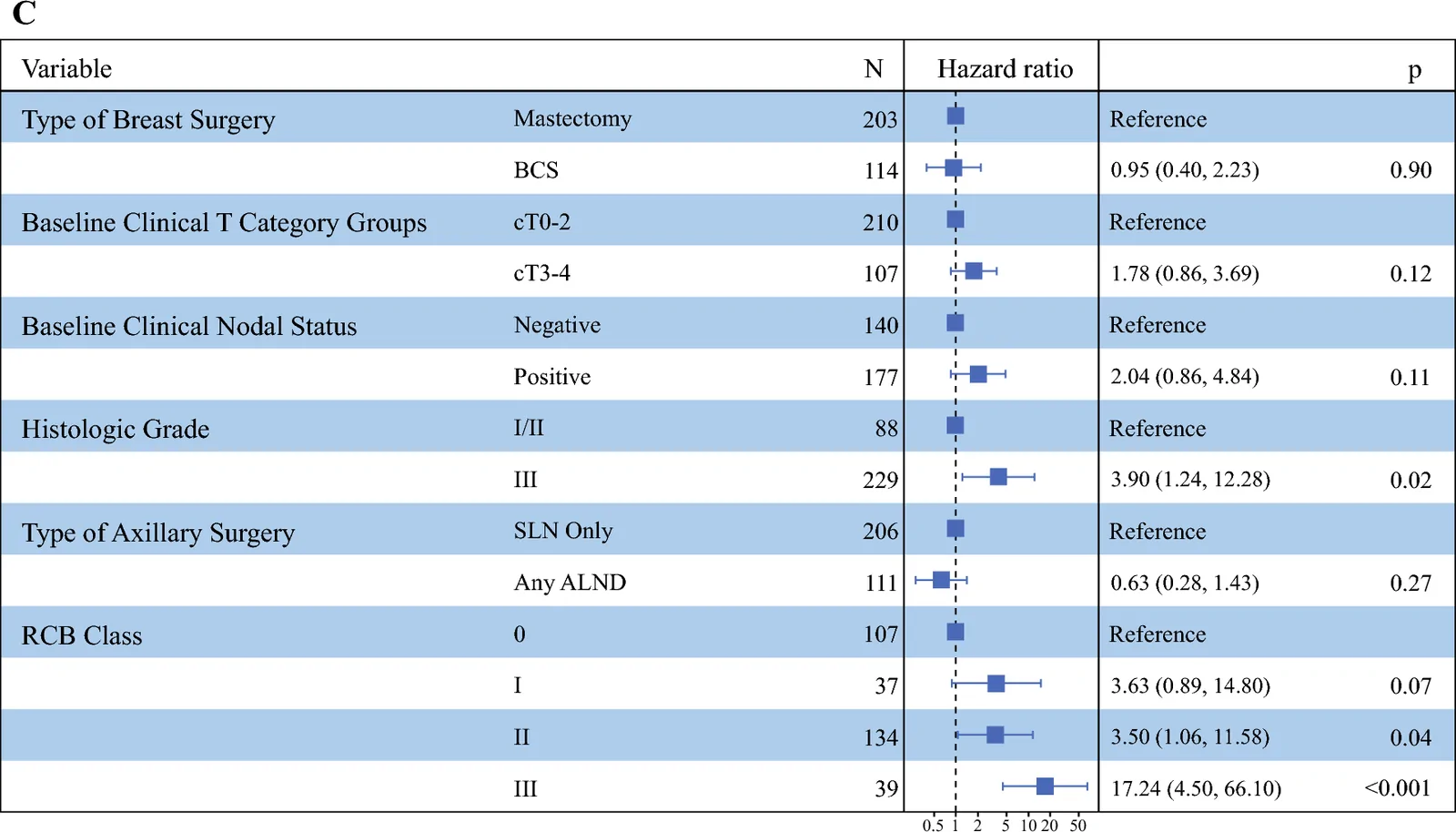
Sub-analysis for patients aged ≤ 40 years. **A** Rates of breast surgery by age group with 40 years as cutoff. Forest plot depicting hazard ratios from Cox proportional hazards model for **B** overall survival (OS) and **C** local recurrence-free survival (LRFI) by breast surgery type and associated factors in patients aged ≤ 40 years. ALND, axillary lymph node dissection; BCS, breast-conserving surgery; HER2, human epidermal growth factor receptor 2; HR+, hormone receptor; RCB, residual cancer burden; SLN, sentinel lymph node surgery; T, tumor; TNBC, triple-negative breast cancer.

In a sub-analysis of patients aged ≤ 40 years (*n* = 444), the rate of women undergoing BCS was 32.4% (*p* < 0.001) (Fig. 6A). Multivariate analysis also did not show a significant difference in OS (HR 0.72; 95% CI 0.32–1.58, *p* = 0.4) (Fig. 6B) or LRFI (HR 0.95; 95% CI 0.40–2.23, *p* =.0.3) (Fig. 6C) by mastectomy or BCS for patients aged ≤ 40 years.

## Discussion

In this analysis of over 1700 women treated with NST, young women aged ≤ 45 years had higher rates of mastectomy; however, locoregional survival and OS was similar for BCS and mastectomy. These results confirm previous findings that young women with early-stage breast cancer who undergo NST have comparable survival outcomes for BCS and mastectomy.

Landmark clinical trials such as NSABP B-06, EORTC 10801, the Milan I Trial, NCI trial, and a meta-analysis by the Early Breast Cancer Trialists' Collaborative Group demonstrated the safety of BCT in younger women.^13–17^ However, these historical studies had generalizability limitations, including predating modern molecular subtype classification, outdated systemic therapy regimens and radiation techniques with late toxicity, limitation in tumor size for clinical trial inclusion, and heterogeneity in surgical technique and margins. Specifically, young patients were excluded or enrolled in low numbers, and the subgroup information about younger women was limited. More recent analyses have also confirmed that BCS and mastectomy are both considered safe surgical treatments for appropriately selected young patients with locally and locoregionally advanced breast cancer, with adjuvant radiation as indicated. Several large database studies found similar LRFI and OS between the two surgical approaches.^1–4,9,18–20^ Most of these studies included patients treated with upfront surgery, with sparse data available for young women undergoing NST. Previously published studies comparing BCS and mastectomy after NST did not primarily focus on the outcomes of young women.^9,20–23^

This study highlights outcomes related to breast surgery choice for young women with high-risk breast cancer who underwent NST. Reassuringly, BCS and mastectomy demonstrated similar OS and LRFI, irrespective of tumor subtype. This study found that younger women enrolled in I-SPY2.0 had similar tumor size and response to NST as older women, suggesting that anatomic candidacy for BCS was similar between younger and older women. Although rates of BCS were lower for the younger age group, notably, there was an increase in use of BCS across the study period of 2010–2022, indicating shifting contemporary breast surgical practices for women aged ≤ 45 years after NST.

Surgical decision-making for young women with breast cancer is complex and involves multiple considerations, and the data available for review were limited. Concern about second breast cancer and ongoing imaging surveillance can influence breast surgery decision-making, and rates of both contralateral prophylactic mastectomy and new contralateral cancers need investigation. Germline pathogenic variants are more common in younger women and could also account for higher rates of mastectomy in this patient population. This study is limited by the lack of germline genetic testing results, although these data are currently being collected across the clinical trial. Individual risk factors significant for prognosis in young women, such as postpartum status, were unavailable for analysis and may have been influential in shared surgical decision-making. Additionally, factors related to patient decision-making relative to surgical recommendations for surgical management were not explicitly collected. Some patients might have selected mastectomy as a means of avoiding radiotherapy, though higher-risk patients such as those in the I-SPY2.0 clinical trial may be more likely to have features prompting the need for postmastectomy radiotherapy.

The choice of 45 years as the primary cutoff to compare age cohorts was made in concordance with other planned I-SPY2.0 clinical trial studies evaluating young women with breast cancer; therefore, a subgroup analysis of women aged < 40 years was conducted to better align with the definition of young women in the existing literature.^1–4^ The clinical relevance of distinguishing < 40 years versus ≤ 45 years may be impactful in thinking about differing psychosocial factors, fertility concerns, and menopausal status. Reassuringly, the subgroup findings for women aged < 40 years were concordant with those aged ≤ 45 years. Importantly, longer-term follow-up will be needed to confirm these results, although clinical and molecular high-risk tumors are known to have the risk of early rather than late recurrence.

In summary, this study demonstrated that young women with early stage, high-risk breast cancer in the I-SPY2.0 clinical trial had lower rates of BCS than older women. The extent of breast surgery did not impact on locoregional recurrence or OS in women aged ≤ 45 years after adjusting for other clinical characteristics and treatment response. Given RCB class was associated with LRFI and OS, tumor biology and response to therapy appear to be more impactful for young women in this study than choice of mastectomy or BCS. The choice of surgical procedure in the management of breast cancer is multifactorial, and young age alone does not necessarily warrant mastectomy following NST.
