# Correction: Surgical Management of Young Women with High-Risk Breast Cancer Receiving Neoadjuvant Systemic Therapy on the I-SPY2 Trial

**DOI:** 10.1245/s10434-026-20526-2

**Published:** 2026-09-03

**Authors:** J. Tseng, K. Dimitroff, C. A. M. Sauder, A. J. Chien, K. A. Ahmed, L. F. Allen, C. A. Arciero, E. E. Burke, J. M. Chandler, J. E. Jordan, M. Golshan, L. A. Gutnik, N. T. Jaskowiak, K. C. Hewitt, M. Howard-McNatt, F. Ito, I. M. Kang, S. B. Kesmodel, H. M. Kuerer, M. C. Lee, M. S. Moran, L. M. Postlewait, N. D. Prionas, R. Rao, C. R. Reyna, A. Schwer, N. K. Taunk, J. Tchou, A. M. Wallace, T. J. Li, J. Perlmutter, A. M. DeMichele, D. Yee, N. M. Hylton, L. J. van’t Veer, H. S. Rugo, W. F. Symmans, R. A. Shatsky, C. J. D. Isaacs, C. Yau, L. J. Esserman, J. C. Boughey, R. A. Mukhtar

**Affiliations:** 1 City of Hope Comprehensive Cancer Center, Irvine, CA USA; 2 Quantum Leap Healthcare Collaborative, San Francisco, CA USA; 3 University of California Davis, Sacramento, CA USA; 4 University of California San Francisco, San Francisco, CA USA; 5 H. Lee Moffitt Cancer Center and Research Institute, Tampa, FL USA; 6 Atrium Health Wake Forest Baptist, Winston-Salem, NC USA; 7 Emory University, Atlanta, GA USA; 8 The Ohio State University Health System, Columbus, OH USA; 9 Yale University School of Medicine, New Haven, CT USA; 10 University of Alabama at Birmingham, Birmingham, AL USA; 11 The University of Chicago Medicine, Chicago, IL USA; 12 University of Utah Health, Salt Lake City, UT USA; 13 University of Southern California, Los Angeles, CA USA; 14 University of Miami Hospital, Miami, FL USA; 15 UT MD Anderson Cancer Center, Houston, TX USA; 16 Columbia University Medical Center, New York, NY USA; 17 Loyola University Medical Center, Maywood, IL USA; 18 University of Pennsylvania, Philadelphia, PA USA; 19 University of California San Diego, San Diego, CA USA; 20 Gemini Group Consultancy, St. Louis, MO USA; 21 University of Minnesota, Minneapolis, MN USA; 22 City of Hope Comprehensive Cancer Center, Duarte, CA USA; 23 Georgetown University, Washington, DC USA; 24 Mayo Clinic, Rochester, MN USA

**Correction to: Annals of Surgical Oncology** **https://doi.org/10.1245/s10434-026-20153-x**

In the original online version of this article, M. S. Moran's middle initial was incorrect. The original article was corrected.

